# Sex differences in congestive markers in patients hospitalized for acute heart failure

**DOI:** 10.1002/ehf2.13300

**Published:** 2021-03-11

**Authors:** Caroline Espersen, Ross T. Campbell, Brian Claggett, Eldrin F. Lewis, John D. Groarke, Kieran F. Docherty, Matthew M.Y. Lee, Moritz Lindner, Tor Biering‐Sørensen, Scott D. Solomon, John J.V. McMurray, Elke Platz

**Affiliations:** ^1^ Cardiovascular Division/Department of Emergency Medicine Brigham and Women's Hospital, Harvard Medical School Boston MA USA; ^2^ BHF Glasgow Cardiovascular Research Centre, Institute of Cardiovascular and Medical Sciences University of Glasgow Glasgow UK; ^3^ The Division of Cardiovascular Medicine Stanford University Medical Center CA USA; ^4^ Department of Cardiology, Herlev and Gentofte Hospital, Faculty of Health Sciences University of Copenhagen Copenhagen Denmark

**Keywords:** Acute heart failure, Lung ultrasound, Congestion, Sex‐specific

## Abstract

**Aims:**

We sought to examine sex differences in congestion in patients hospitalized for acute heart failure (AHF). Understanding congestive patterns in women and men with AHF may provide insights into sex differences in the presentation and prognosis of AHF patients.

**Methods and results:**

In a prospective, two‐site study in adults hospitalized for AHF, four‐zone lung ultrasound (LUS) was performed at the time of echocardiography at baseline (LUS1) and, in a subset, pre‐discharge (LUS2). B‐lines on LUS and echocardiographic images were analysed offline, blinded to clinical information and outcomes. Among 349 patients with LUS1 data (median age 74, 59% male, and 87% White), women had higher left ventricular ejection fraction (mean 43% vs. 36%, *P* < 0.001), higher tricuspid annular plane systolic excursion (mean 17 vs. 15 mm, *P* = 0.021), and higher measures of filling pressures (median E/e′ 20 vs. 16, *P* < 0.001). B‐line number on LUS1 (median 6 vs. 6, *P* = 0.69) and admission N‐terminal pro‐B‐type natriuretic peptide levels (median 3932 vs. 3483 pg/mL, *P* = 0.77) were similar in women and men. In 121 patients with both LUS1 and LUS2 data, there was a similar and significant decrease in B‐lines from baseline to discharge in both women and men. The risk of the composite 90 day outcome increased with higher B‐line number on four‐zone LUS2: unadjusted hazard ratio for each B‐line tertile was 1.86 (95% confidence interval 1.08–3.20, *P* = 0.025) in women and 1.65 (95% confidence interval 1.03–2.64, *P* = 0.037) in men (interaction *P* = 0.72).

**Conclusions:**

Among patients with AHF, echocardiographic markers differed between women and men at baseline, whereas B‐line number on LUS did not. The dynamic changes in B‐lines during a hospitalization for AHF were similar in women and men.

## Introduction

Several sex differences have been reported in patients with heart failure (HF) with respect to clinical presentation, risk factors, pathophysiology, and prognosis.^1^ Although these important sex differences have been previously investigated in patients with HF, little is known about the sex differences in pulmonary congestion on lung ultrasound (LUS) in patients with HF. Congestion, particularly pulmonary congestion, is a cardinal finding in patients with HF affecting the presentation and prognosis of these patients. Based on data from ADHERE and PROTECT, women with acute HF (AHF) demonstrate more pronounced pulmonary congestion at baseline with a higher prevalence of dyspnoea,^2^ orthopnoea,^3^ and rales.^2^, ^3^ Although the differences are subtle in the ADHERE registry, it is conceivable that LUS findings as a more sensitive measure of pulmonary congestion in AHF may also differ between women and men.

Understanding the congestive patterns in women and men may provide insights into the differences in presentation and prognosis and thus aid in better patient management and risk stratification. We, therefore, sought to investigate the sex differences in congestive markers based on the physical examination, chest X‐ray (CXR), echocardiography, laboratory tests, and LUS in patients hospitalized for AHF. A secondary aim was to examine the dynamic changes of congestive markers during a hospitalization for AHF and in exploratory analyses the prognostic value of these markers at discharge.

## Methods

### Study population

This is a *post hoc* analysis of a prospective, two‐centre observational study in adult patients hospitalized for AHF at two academic hospitals in Boston (USA) and in Glasgow (UK). Details regarding the study population have previously been described.^4^ Briefly, patients were enrolled from inpatient units (both sites) and emergency department observation units (Boston) if they were admitted with a diagnosis of AHF (presenting with HF signs and symptoms and requiring intravenous diuretics irrespective of ejection fraction). Patients were excluded from this study if they presented with important lung diseases potentially affecting LUS findings (e.g. pulmonary fibrosis and pneumonia), isolated right HF, pregnancy, B‐type natriuretic peptide <100 pg/mL in Glasgow and N‐terminal pro‐B‐type natriuretic peptide (NT‐proBNP) <1400 pg/mL in Boston, or current dialysis. Lastly, for this analysis, only patients who had an LUS performed at baseline (LUS1) were included (Supporting Information, *Figure* *S1*). LUS and echocardiography were performed early during hospitalization within a median of 1 day after admission in both Glasgow (interquartile range 1–2 days) and Boston (inter‐quartile range 1–1 day). Patients gave written informed consent prior to participation. This study complied with the Declaration of Helsinki and was approved by the local institutional review boards (Partners IRB and the West of Scotland Research Ethics Service). The first and senior authors had full access to all the data in the study and take responsibility for its integrity and the data analysis.

### Congestive markers

#### Lung ultrasonography protocol and image analysis

Lung ultrasound examinations were performed by trained investigators using conventional echocardiographic equipment with a phased array transducer at baseline (LUS1) and in a subset at discharge (LUS2). A simplified four‐zone imaging protocol was used for LUS assessment at both sites, and in addition, an eight‐zone LUS examination was performed in Boston. The LUS images were analysed offline by experienced investigators blinded to clinical data, timing of LUS, and outcomes as previously described.^4^ The sum of the maximum number of B‐lines from each zone was used to obtain the total number of B‐lines for each patient. With regard to the intra‐reader and inter‐reader agreement, the mean B‐line difference in 25 patients for the four‐zone LUS image analysis was 0.4 (95% limits of agreement −1.1, 2.0) and 1.1 (95% limits of agreement −2.2, 4.6) in Bland–Altman analyses, respectively.^4^

#### Assessment of pleural effusions

The assessment of pleural effusion was performed at baseline and discharge using the same equipment as for the LUS examination with the transducer positioned laterally at the level of the diaphragm on each hemithorax with patients in a semi‐recumbent position as previously described.^5^ The presence of pleural effusions was quantified for each hemithorax using the semi‐quantitative pleural effusion score ranging from 0 to 4 points.^5^ The total pleural effusion score for each patient was then calculated as the sum of the pleural effusion score from each hemithorax with values ranging from 0 to 8.

#### Echocardiography

All patients underwent a conventional echocardiographic examination early during the hospitalization. Images were acquired using conventional echocardiographic equipment with phased array transducers. Images were analysed offline following the American Society of Echocardiography guidelines^6^ by trained investigators at each site who were blinded to clinical data and long‐term outcomes. Left ventricular ejection fraction (LVEF) was obtained using Simpson's biplane method. HF with preserved ejection fraction (HFpEF) was defined as LVEF ≥ 45%. Left ventricular mass index was calculated using linear left ventricular measurements and indexed to body surface area. Left atrial volume index was calculated using the area–length method and indexed to body surface area. Peak velocity of early (E) and late (A) diastolic filling was measured with pulsed‐wave Doppler in the apical four‐chamber view with the sample volume placed between the mitral leaflet tips, and the E/A ratio was calculated. Tissue Doppler imaging was used to obtain the average annular mitral peak early diastolic velocity (e′) from the lateral and septal e′ values, and the E/e′ ratio was calculated from the average lateral and septal e′ measurements. Tricuspid annular plane systolic excursion (TAPSE) was measured by M‐mode tracings of the tricuspid annulus from an apical four‐chamber view focused on the right ventricle (RV). Tricuspid regurgitation velocity was measured from the tricuspid regurgitation jet using continuous‐wave Doppler in the RV apical four‐chamber view. The estimated systolic pulmonary artery pressure was calculated from the tricuspid regurgitation velocity using the Bernoulli equation. Myocardial systolic excursion velocity, s′, was measured at the lateral tricuspid annulus using tissue Doppler imaging in the apical four‐chamber view. The maximum inferior vena cava (IVC) diameter was measured 1–2 cm proximal to the junction of the IVC and the right atrium.^6^ The RV fractional area change was calculated from the end‐diastolic and end‐systolic areas of the RV in the apical four‐chamber view.

#### Chest X‐ray on admission

If clinically indicated, patients underwent an upright CXR examination on admission. These CXRs were analysed by radiologists for the presence of vascular congestion, interstitial oedema, and alveolar oedema.^5^ Only CXR examinations performed within 1 day of admission based on date change were included in this analysis.

#### Physical examination, biochemical tests, and clinical and demographic data

Laboratory tests were performed at baseline at both sites, and clinical and demographic data were collected from medical records by trained investigators at each site. Dyspnoea assessments were completed by each patient at the time of LUS1 and LUS2 based on a numerical ranking scale ranging from 0 to 10 with 0 corresponding to no dyspnoea while lying flat and 10 corresponding to severe dyspnoea at rest.^7^, ^8^

### Outcomes

The endpoint was time‐to‐first event of the composite outcome of HF readmission or all‐cause mortality at 90 days after discharge. Because of a differential association of B‐lines and the outcome over time, in which the relationship between B‐lines and the primary outcome was strongest early after discharge and attenuated over time, we chose to analyse the outcome at 90 days.^4^ HF hospitalizations were adjudicated by experienced physicians at each site and verified through patient follow‐up phone calls, contacting primary care physicians or cardiologists, and review of electronic medical records. All‐cause mortality was confirmed through review of electronic medical records at both sites and review of social security index and obituaries in Boston. We assessed the outcomes in patients with an available LUS2 (*n* = 132). As a sensitivity analysis, we also examined outcomes in the LUS1 cohort (*n* = 339). Ten patients were excluded from the LUS1 cohort in the outcome analysis as they died or had a left ventricular assist device implanted during the initial hospital stay (CONSORT diagram, Supporting Information, *Figure* *S1*).

### Statistical analyses

Baseline characteristics and congestive markers were summarized in women and men. Categorical variables were compared using the *χ* ^2^ test or Fisher's exact test as appropriate. Continuous variables were compared using Student's *t*‐test or Wilcoxon rank‐sum test as appropriate. We investigated the correlation between the number of B‐lines on four‐zone LUS1 and the log NT‐proBNP levels using Spearman's correlation coefficient.

Dynamic changes in congestive markers from baseline to pre‐discharge were analysed using Student's paired *t*‐test or Wilcoxon signed‐rank test for comparing paired continuous observations as appropriate. McNemar's test and McNemar's exact test were used to compare paired categorical observations from baseline to pre‐discharge as appropriate. To compare the difference in the change in continuous markers between women and men, we used Student's *t*‐test and Wilcoxon rank‐sum test.

Unadjusted and adjusted Cox proportional hazards models were used to assess the risk of the composite endpoint of rehospitalization for HF or all‐cause mortality in women vs. men. In Model 1, we adjusted for age, LVEF, baseline log creatinine, and baseline systolic blood pressure, stratified by study site. In Model 2, we adjusted for the same variables, but instead of LVEF, we included log NT‐proBNP and stratified by study site. To further analyse possible predictors of the 90 day composite outcome in women and men separately, unadjusted Cox proportional hazards model was used to investigate the prognostic value of available congestive markers at discharge on the 90 day composite outcome. Results of these analyses were considered exploratory and hypothesis generating. The proportional hazards assumptions were assessed using Schoenfeld residuals, and interaction between sex and each covariate in the model was also checked. Lastly, in order to examine whether the baseline or discharge congestive marker was the most important predictor of the composite outcome independent of sex, we included both the baseline and corresponding discharge congestive marker in the same model adjusted for sex. Statistical analyses were performed using Stata SE, Version 14.1 (StataCorp., College Station, TX, USA, 2015), and a two‐sided *P*‐value < 0.05 was considered statistically significant.

## Results

### Baseline demographic and clinical parameters in women and men

Of 370 eligible patients, 349 patients had an LUS examination performed at baseline (LUS1) and were thus included in this analysis (Glasgow *n* = 157; Boston *n* = 192). In this patient cohort, there were 144 (41%) female patients. Baseline characteristics of women and men are outlined in *Table* *1*. Overall, women were older and had a lower body mass index and higher systolic blood pressure. Women more often had HFpEF compared with men. Men were more likely to have a history of prior HF, HF hospitalizations, and coronary heart disease, whereas women were more likely to have a history of depression. At baseline, men were more often treated with beta‐blockers and diuretics.

**Table 1:** Baseline characteristics of women and men (*n* = 349)

	*n*	Women (*n* = 144)	Men (*n* = 205)	*P*
**Demographics**				
Age (years)	349	76 [70, 83]	73 [64, 81]	0.011
Hispanic, *n* (%)	191	6 (8%)	5 (4%)	0.34
Non‐Hispanic, White, *n* (%)	349	120 (83%)	183 (89%)	0.11
Non‐Hispanic, Black, *n* (%)	349	17 (12%)	15 (7%)	0.15
**Clinical characteristics**				
BMI (kg/m^2^)	345	26 [22, 31]	28 [24, 32]	0.018
Heart rate (b.p.m.)	347	85 ± 22	81 ± 21	0.10
Systolic blood pressure (mmHg)	347	134 ± 28	125 ± 26	0.004
Diastolic blood pressure (mmHg)	346	72 ± 16	70 ± 16	0.31
NYHA functional class	349			0.07
I and II		33 (23%)	70 (34%)	
III		83 (58%)	97 (47%)	
IV		28 (19%)	38 (19%)	
HFpEF (LVEF ≥ 45%), *n* (%)	349	67 (47%)	57 (28%)	<0.001
**Medical history**				
Prior HF, *n* (%)	349	74 (51%)	144 (70%)	<0.001
Prior HF hospitalization, *n* (%)	349	49 (34%)	103 (50%)	0.003
Hypertension, *n* (%)	349	117 (81%)	153 (75%)	0.15
Diabetes, *n* (%)	349	48 (33%)	83 (40%)	0.17
Atrial fibrillation/flutter, *n* (%)	349	71 (49%)	121 (59%)	0.07
COPD, *n* (%)	349	32 (22%)	37 (18%)	0.34
PCI, *n* (%)	349	22 (15%)	50 (24%)	0.038
CABG, *n* (%)	349	19 (13%)	61 (30%)	<0.001
Myocardial infarction, *n* (%)	349	39 (27%)	86 (42%)	0.004
Pacemaker, *n* (%)	349	17 (12%)	32 (16%)	0.22
CRT, *n* (%)	349	6 (4%)	19 (9%)	0.07
Depression, *n* (%)	347	28 (20%)	24 (12%)	0.045
**Home medications**				
Beta‐blockers, *n* (%)	349	95 (66%)	156 (76%)	0.038
ACE inhibitor/ARB, *n* (%)	349	67 (47%)	103 (50%)	0.49
ARNI, *n* (%)	192	2 (3%)	2 (2%)	0.63
Aldosterone blocker, *n* (%)	349	13 (9%)	31 (15%)	0.09
Diuretic(s), *n* (%)	349	83 (58%)	143 (70%)	0.02
Digoxin, *n* (%)	349	15 (10%)	16 (8%)	0.40
Calcium channel blockers, *n* (%)	349	39 (27%)	39 (19%)	0.08
Amiodarone, *n* (%)	197	8 (11%)	19 (16%)	0.40
Insulin, *n* (%)	349	23 (16%)	40 (20%)	0.56
Anticoagulation, *n* (%)	349	53 (37%)	91 (44%)	0.16

ACE, angiotensin‐converting enzyme; ARB, angiotensin receptor blocker; ARNI, angiotensin receptor neprilysin inhibitor; BMI, body mass index; b.p.m., beats per minute; CABG, coronary artery bypass graft; COPD, chronic obstructive pulmonary disease; CRT, cardiac resynchronization therapy; HF, heart failure; HFpEF, heart failure with preserved ejection fraction; LVEF, left ventricular ejection fraction; NYHA, New York Heart Association; PCI, percutaneous coronary intervention.

### Baseline congestive markers

Findings related to congestion are outlined in *Table* *2*. Women reported significantly more severe dyspnoea than men at baseline (median score 5 vs. 3, *P* < 0.001). On physical examination, men were more likely than women to present with lower extremity oedema on admission (*P* = 0.011). There were also significant sex differences in the echocardiographic measures. Overall, women had a better left and right systolic function with a higher LVEF (mean 43% vs. 36%, *P* < 0.001) and higher TAPSE (mean 17 vs. 15 mm, *P* = 0.021) and RV fractional area change (mean 26% vs. 22%, *P* = 0.012), but greater measures of filling pressures (E/e′ ratio median 19.5 vs. 15.8, *P* < 0.001) and higher tricuspid regurgitation velocity (mean 2.98 vs. 2.84 m/s, *P* = 0.039) than men. By contrast, men had a higher E/A ratio (mean 2.12 vs. 1.46, *P* < 0.001) and a greater maximum IVC diameter (2.3 vs. 2.1 cm, *P* < 0.001). The distribution of pleural effusion scores in tertiles on LUS was significantly different between women and men, with women presenting with higher pleural effusion scores, although the median pleural effusion score was not significantly different between women and men. The median number of B‐lines and the distribution of B‐lines in tertiles on LUS1 were not significantly different between women and men. These findings were similar in HFpEF (n = 124) and HF with reduced ejection fraction (n = 225). The laboratory tests revealed better renal function in women compared with men. The NT‐proBNP levels were not significantly different between women and men, and there was a similar, although weak, positive correlation between log (NT‐proBNP) levels and number of B‐lines on four‐zone LUS1 in both women and men (*r* = 0.36, *P* < 0.001 and *r* = 0.23, *P* = 0.002, respectively) (Supporting Information, *Figure* *S2*).

**Table 2:** Congestive markers and length of hospital stay in women and men (*n* = 349)

	*n*	Women (*n* = 144)	Men (*n* = 205)	*P*
**Dyspnoea score (range 0–10; 10 worst)**				
Dyspnoea at rest	190	5 [3, 8]	3 [0, 7]	<0.001
**Baseline laboratory results**				
NT‐proBNP (pg/mL)	316	3483 [1841, 7971]	3932 [1794, 7927]	0.77
Sodium (mmol/L)	349	138 [135, 141]	138 [136, 140]	0.45
Potassium (mmol/L)	346	4.21 ± 0.60	4.26 ± 0.62	0.37
Haemoglobin (g/dL)	349	11.5 ± 1.9	11.9 ± 2.3	0.09
BUN (mg/dL)	349	22 [17, 33]	28 [21, 50]	<0.001
Creatinine (mg/dL)	349	1.1 [0.8, 1.5]	1.4 [1.1, 2.1]	<0.001
Albumin (g/dL)	320	3.45 ± 0.48	3.51 ± 0.46	0.22
**Baseline CXR** ^a^				
Vascular congestion, *n* (%)	138	42 (71%)	55 (70%)	0.84
Interstitial oedema, *n* (%)	139	52 (87%)	62 (78%)	0.27
Alveolar oedema, *n* (%)	139	9 (15%)	9 (11%)	0.61
**Echocardiography**				
LVEF (%)	349	43 ± 16	36 ± 15	<0.001
LVMI (g/m^2^)	318	113 ± 34	128 ± 41	0.005
LAVI (mL/m^2^)	335	47 [33, 63]	44 [36, 56]	0.66
E (m/s)	299	1.14 ± 0.35	1.03 ± 0.31	0.004
A (m/s)	176	0.77 [0.53, 1.01]	0.49 [0.36, 0.72]	<0.001
E/A	173	1.46 [0.92, 2.10]	2.12 [1.40, 2.83]	<0.001
e′, cm/s (average)	297	0.06 ± 0.02	0.06 ± 0.02	0.025
E/e′ (average)	286	19.5 [15.2, 26.3]	15.8 [12.9, 20.5]	<0.001
TAPSE (mm)	285	17 ± 6	15 ± 6	0.021
TR velocity (m/s)	317	2.98 ± 0.63	2.84 ± 0.57	0.039
s′ (cm/s)	329	0.07 [0.05, 0.10]	0.08 [0.05, 0.10]	0.16
IVC max (cm)	300	2.1 ± 0.54	2.3 ± 0.52	<0.001
Estimated systolic PA pressure (mmHg)	170	47 ± 18	45 ± 15	0.57
RV fractional area change (%)	157	26 ± 10	22 ± 9	0.012
**Physical examination**				
Weight (kg)	345	66 [57, 80]	84 [74, 98]	<0.001
SpO_2_ (%)	346	96 [95, 98]	97 [95, 98]	0.26
Supplemental O_2_, *n* (%)	344	47 (33%)	51 (25%)	0.11
Jugular venous distension (>10 cm), *n* (%)	177	45 (70%)	91 (81%)	0.12
Crackles on auscultation, *n* (%)	349	101 (70%)	125 (61%)	0.08
Lower extremity oedema, *n* (%)	349	98 (68%)	164 (80%)	0.011
S3, *n* (%)	347	25 (18%)	30 (15%)	0.49
**LUS1**				
Number of B‐lines, 4 zones (tertiles), *n* (%)	349			0.96
0–4 B‐lines		51 (35%)	70 (34%)	
5–9 B‐lines		54 (38%)	77 (38%)	
≥10 B‐lines		39 (27%)	58 (28%)	
B‐line count, 4 zones	349	6 [3, 10]	6 [3, 10]	0.69
Number of B‐lines, 8 zones (tertiles), *n* (%)	192			0.24
0–5 B‐lines		17 (23%)	29 (25%)	
6–11 B‐lines		25 (34%)	27 (23%)	
≥12 B‐lines		32 (43%)	62 (53%)	
B‐line count, 8 zones, *n* (%)	192	10 [6, 16]	12 [6, 19]	0.13
Pleural effusion score (tertiles), *n* (%)	178			0.048
0		15 (23%)	42 (37%)	
1–4		27 (42%)	29 (25%)	
5–8		22 (34%)	43 (38%)	
Pleural effusion score	178	2 [1, 6]	2 [0, 6]	0.29
**Length of stay (days)**	349	6 [3, 13]	7 [3, 12]	0.95

A, late mitral inflow velocity; BUN, blood urea nitrogen; CXR, chest X‐ray; E, early mitral inflow velocity; E/A, ratio between early mitral inflow velocity and late mitral inflow velocity; E/e′, ratio between early mitral inflow velocity and mitral annular early diastolic velocity; e′, mitral annular early diastolic velocity; IVC, inferior vena cava; LAVI, left atrial volume index; LUS1, lung ultrasound at baseline; LVEF, left ventricular ejection fraction; LVMI, left ventricular mass index; NT‐proBNP, N‐terminal pro‐B‐type natriuretic peptide; PA, pulmonary artery; RV, right ventricle; s′, myocardial systolic excursion velocity; S3, third heart sound; SpO_2_, blood oxygen level; supplemental O_2_, supplemental oxygen; TAPSE, tricuspid annular plane systolic excursion; TR, tricuspid regurgitation.

^a^ Within 1 day of admission.

### Dynamic changes in congestive markers from baseline to discharge

In 129 patients with both LUS1 and LUS2, the dyspnoea score at rest, several physical examination parameters, blood urea nitrogen values, and LUS findings differed significantly from baseline to discharge in women and men (*Table* *3*).

**Table 3:** Dynamic changes in congestive markers from baseline to pre‐discharge in women and men (*n* = 121)

Congestion marker	Women (*n* = 46)					Men (*n* = 75)					Difference in change between women and men
	Obs. (*n*)	Baseline	Pre‐discharge	Change	*P*	Obs. (*n*)	Baseline	Pre‐discharge	Change	*P*	*P*
**Dyspnoea score (range 0–10; 10 worst)**											
Dyspnoea at rest	45	6 [3, 8]	3 [1, 6]	−2 ± 3	<0.001	73	2 [0, 6]	1 [0, 3]	−1 ± 3	<0.001	0.44
**Physical exam**											
Weight (kg)	45	72 [55, 92]	69 [56, 86]	−2.3 ± 4.2	<0.001	74	85 [73, 101]	80 [69, 98]	−3.8 ± 4.2	<0.001	0.06
SpO_2_ (%)	45	96 [95, 98]	96 [94, 98]	−0.2 ± 2.9	0.67	75	96 [94, 98]	97 [96, 98]	0.5 ± 2.2	0.13	0.16
Supplemental O_2_, *n* (%)	45	19 (42%)	5 (11%)	—	0.001	75	20 (27%)	5 (7%)	—	0.002	—
JVD > 10 cm, *n* (%)	35	24 (69%)	4 (11%)	—	<0.001	65	52 (80%)	9 (14%)	—	<0.001	—
Crackles (any), *n* (%)	44	23 (52%)	20 (45%)	—	0.58	72	44 (61%)	23 (32%)	—	<0.001	—
Lower extremity oedema (any), *n* (%)	43	21 (49%)	24 (56%)	—	0.47	70	55 (79%)	40 (57%)	—	0.001	—
S3, *n* (%)	44	1 (2%)	0 (0%)	—	1.00	73	7 (10%)	5 (7%)	—	0.63	—
**Laboratory values**											
Sodium (mmol/L)	44	139 [136, 141]	139 [136, 141]	0.1 ± 5	0.82	75	139 [136, 141]	138 [136, 141]	0.4 ± 4	0.73	0.72
BUN (mg/dL)	44	22 [17, 31]	29 [21, 47]	9 ± 14	<0.001	75	35 [22, 58]	35 [25, 65]	5 ± 17	0.008	0.30
Creatinine (mg/dL)	44	1.1 [0.8, 1.6]	1.2 [0.9, 1.6]	0.03 ± 0.37	0.47	75	1.6 [1.2, 2.7]	1.6 [1.2, 2.4]	−0.05 ± 0.39	0.12	0.27
**Lung ultrasound**											
B‐line count (4 zones)	46	6 [3, 8]	3 [1, 7]	−1 ± 4	0.014	75	6 [4, 10]	4 [2, 7]	−2 ± 4	<0.001	0.36
B‐line count (8 zones)	45	10 [6, 15]	7 [3, 12]	−3 ± 6	<0.001	75	14 [7, 21]	9 [5, 13]	−4 ± 7	<0.001	0.46
Pleural effusion score	36	2.5 [1, 6]	0 [0, 3]	−1 [−2.5, 0]	<0.001	68	2 [0, 6]	0.5 [0, 4]	0 [−2, 0]	<0.001	0.13

BUN, blood urea nitrogen; JVD, jugular venous distension; S3, third heart sound; SpO_2_, blood oxygen level; supplemental O_2_, supplemental oxygen.

**Figure 1:**
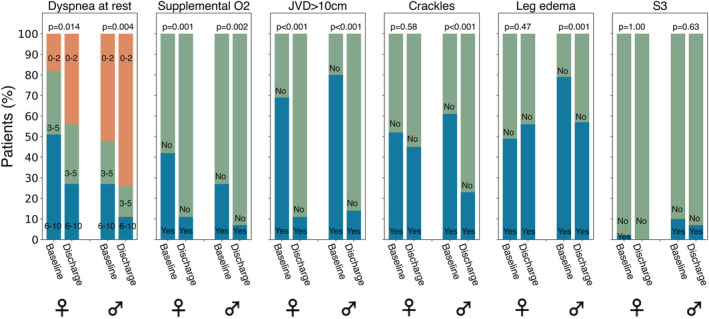
Dynamic changes in physical examination parameters and dyspnoea score from baseline to discharge (*n* = 121) in women (*n* = 46) and men (*n* = 75). Dyspnoea score at rest divided into three categories: blue represents a score of 6‐10, green represents a score of 3–5, and orange represents a score of 0‐2. For the physical examination parameters, blue represents presence of the particular physical examination parameter, and green represents absence of the particular physical examination parameter. JVD, jugular venous distension.

**Figure 2:**
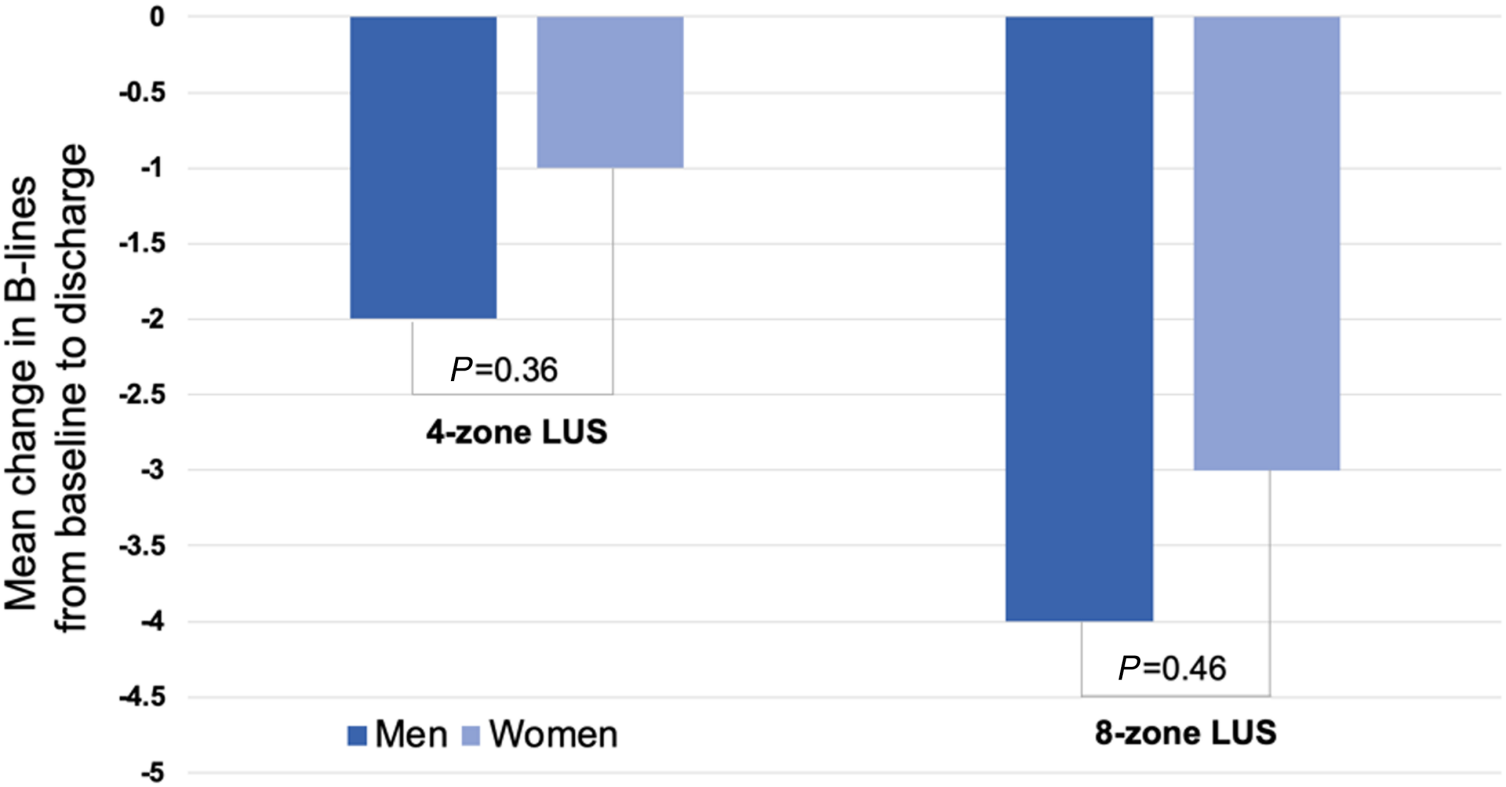
Dynamic changes in B‐lines from baseline to discharge on the four‐zone and eight‐zone lung ultrasound (LUS) in women and men (*n* = 121). Four‐zone LUS: change in B‐lines from baseline to discharge in women (*n* = 46), *P* = 0.014, and in men (*n* = 75), *P* < 0.001. Eight‐zone LUS: change in B‐lines from baseline to discharge in women (*n* = 45), *P* < 0.001, and in men (*n* = 75), *P* < 0.001.

Although there were significant differences in these congestive markers from baseline to discharge in women and men separately, the change in the continuous congestive markers from baseline to discharge was not significantly different between women and men. However, while the proportion of women with crackles on auscultation and lower extremity oedema did not change significantly from baseline to discharge, these proportions decreased significantly from baseline to discharge in men (*Figure* *1*). The change in B‐lines from both four‐zone and eight‐zone LUS from baseline to discharge did not differ significantly between women and men (*Figure* *2*).

### Ninety day outcomes in women and men

Considering the 132 patients who underwent a discharge LUS examination (LUS2, *n* = 132), 17 (32.1%) women and 25 (31.6%) men experienced the composite outcome of all‐cause death or HF hospitalization during the 90 day follow‐up (*P* = 0.96) (*Table* *4*). There were no significant differences in 90 day outcomes between women and men with available LUS2 data. In sensitivity analyses, we compared event rates in the entire cohort of patients with LUS1 data and the subset of patients with LUS2 data (*Table* *4*). Among the 339 patients with an LUS1 who were not censored prior to hospital discharge, 37 (25.9%) women and 49 (25.0%) men experienced the composite outcome over the 90 day follow‐up period (*P* = 0.86). There were also no significant differences in 90 day HF hospitalization, all‐cause death, or composite outcome in women and men in the LUS1 cohort.

**Table 4:** Ninety day outcomes by sex in patients with LUS1 (*n* = 339) and patients with LUS2 (*n* = 132)

Outcome	LUS1 cohort (*n* = 339): 90 day outcomes^a^			LUS2 cohort (*n* = 132): 90 day outcomes		
	Men (*n* = 196)	Women (*n* = 143)	*P*	Men (*n* = 79)	Women (*n* = 53)	*P*
HF hospitalization	36 (18%)	32 (22%)	0.36	18 (23%)	14 (26%)	0.63
All‐cause death	16 (8%)	11 (8%)	0.87	8 (10%)	5 (9%)	0.90
Composite	49 (25%)	37 (26%)	0.86	25 (32%)	17 (32%)	0.96

HF, heart failure; LUS1, lung ultrasound at baseline; LUS2, lung ultrasound at discharge.

^a^ Ten patients from the LUS1 cohort had in‐hospital deaths or a left ventricular assist device implanted; therefore, they are excluded for the outcome analysis.

### Predictors of 90 day outcomes and interaction with sex

The risk of the composite 90 day outcome in women compared with men was not significantly different in unadjusted or adjusted models (Supporting Information, *Table* *S1*).

In an exploratory analysis, we investigated the predictive value of discharge congestive parameters for the 90 day composite outcome in women and men separately in univariable analysis (*Table* *5*). No significant violations of the proportional hazards assumptions were detected. The risk of the composite 90 day outcome increased with higher number of B‐lines on four‐zone LUS2 in both women and men [unadjusted hazard ratio (HR) for each B‐line tertile in women 1.86, 95% confidence interval (CI) 1.08–3.20, *P* = 0.025 and in men 1.65, 95% CI 1.03–2.64, *P* = 0.037; interaction *P* = 0.72]. In men, dyspnoea scores at rest (HR 1.17, 95% CI 1.01–1.36, *P* = 0.032), blood urea nitrogen values (HR 1.02, 95% CI 1.00–1.03, *P* = 0.011), and number of B‐lines on eight‐zone LUS2 (HR 1.72, 95% CI 1.01–2.92, *P* = 0.046) were also significantly associated with the risk of the composite 90 day outcome in univariable analysis. None of the other available congestive markers at discharge were significant predictors of the 90 day composite outcome in women or men.

**Table 5:** Predictors of composite outcome at 90 days using congestive markers at discharge (*n* = 132)

Variables	At 90 days (*n* = 132)				
	Women (*n* = 53)		Men (*n* = 79)		Interaction with sex
Unadjusted	HR (95% CI)	*P*	HR (95% CI)	*P*	*P*
**Dyspnoea score (range 0–10; 10 worst)**					
Dyspnoea at rest (per 1 score increase)	1.11 (0.97–1.28)	0.14	1.17 (1.01–1.36)	0.032	0.65
**Physical exam** ^a^					
Weight (per 1 kg increase)	0.99 (0.97–1.01)	0.40	0.99 (0.97–1.01)	0.24	0.87
SpO_2_ (per 1% increase)	1.13 (0.91–1.39)	0.27	0.96 (0.79–1.17)	0.70	0.28
Supplemental O_2_	1.44 (0.41–5.06)	0.57	1.18 (0.28–5.01)	0.82	0.85
JVD > 10 cm	2.11 (0.47–9.46)	0.33	1.67 (0.57–4.90)	0.36	0.82
Crackles (any)	1.14 (0.43–3.04)	0.79	1.25 (0.55–2.84)	0.59	0.90
Lower extremity oedema (any)	0.51 (0.25–1.04)	0.07	1.41 (0.71–2.82)	0.33	0.98
**Laboratory values**					
Sodium (per 1 mmol/L increase)	0.99 (0.86–1.15)	0.90	0.94 (0.84–1.04)	0.24	0.52
BUN (per 1 mg/dL increase)	1.01 (0.99–1.03)	0.60	1.02 (1.00–1.03)	0.011	0.42
Creatinine (per 1 mg/dL increase)	1.22 (0.68–2.17)	0.50	1.25 (0.97–1.60)	0.08	0.95
**Lung ultrasound**					
Number of B‐lines, 4 zones (per tertile increase, trend)^b^	1.86 (1.08–3.20)	0.025	1.65 (1.03–2.64)	0.037	0.72
Number of B‐lines, 8 zones (per tertile increase, trend)^c^	1.64 (0.90–2.99)	0.11	1.72 (1.01–2.92)	0.046	0.95
Pleural effusion score at time of LUS2 (per tertile increase, trend)^d^	1.63 (0.86–3.12)	0.14	0.97 (0.57–1.65)	0.91	0.22

BUN, blood urea nitrogen; JVD, jugular venous distension; LUS2, lung ultrasound at discharge; SpO_2_, blood oxygen level; supplemental O_2_, supplemental oxygen.

^a^ S3 was not included in the outcome analysis, as there were too few measurements for women.

^b^ B‐line tertiles for four‐zone LUS: 0–3, 4–6, and ≥7 B‐lines.

^c^ B‐line tertiles for eight‐zone LUS: 0–5, 6–11, and ≥12 B‐lines.

^d^ Pleural effusion score tertiles; 0, 1–4, and 5–8.

When including both the baseline LUS1 and the discharge LUS2 from four‐zone and eight‐zone LUS in the same Cox regression model, only the discharge LUS2 was a significant predictor of outcomes when adjusting for sex (Supporting Information, *Table* *S2*). Similarly, for the dyspnoea score at rest, only the discharge score was a significant predictor of outcome. However, for the other congestive markers, neither the baseline nor the discharge markers were significant predictors.

## Discussion

In this prospective, two‐centre study, we investigated sex differences in congestive markers based on several diagnostic examinations in patients hospitalized for AHF. Despite similar radiological findings and NT‐proBNP levels, women hospitalized for AHF were significantly more dyspnoeic than men and had a substantially higher E/e′ than men at baseline. Conversely, men more often had lower extremity oedema and a higher maximum IVC diameter than women at baseline. Despite this different presentation, the number of B‐lines on baseline LUS did not differ between women and men. However, as expected, more women than men had HFpEF (47% vs. 28%, *P* < 0.001).

### Baseline congestive markers and dynamic changes

Similar to prior studies in patients with AHF,^2^, ^3^, ^9^, ^10^, ^11^, ^12^, ^13^, ^14^ women were older and had higher systolic blood pressure in this cohort. The baseline LUS findings were similar in women and men and consistent with the baseline CXR findings, with respect to the presence of vascular congestion, interstitial oedema, or alveolar oedema, and, likewise, findings on lung auscultation. Despite these consistent findings regarding pulmonary congestion in both sexes, women reported worse dyspnoea and had higher measures of filling pressures (E/e′) at baseline. These findings could indicate differences in haemodynamics between women and men with AHF and highlight the non‐specific nature of dyspnoea in this disease state. Our findings indicate further that pulmonary congestion responds similarly to HF therapy during an AHF hospitalization in both sexes as measured by the reduction in B‐lines on LUS. Furthermore, the correlation between B‐lines and log NT‐proBNP levels in women and men was similar and significant. These findings suggest that the prevalence and dynamic changes in LUS findings in women and men with AHF are similar and that sex‐specific cut‐off values may not be needed.

During the hospitalization, there was a similar and significant decrease in the number of B‐lines and pleural effusion size from baseline to discharge in both women and men. Although women were more dyspnoeic than men at baseline with higher dyspnoea scores on a numeric ranking scale, the decrease in dyspnoea score from baseline to discharge was similar in women and men. However, whereas women did not display a significant decrease in the proportion of patients with crackles on auscultation or lower extremity oedema from baseline to discharge, men did. A higher proportion of men had lower extremity oedema at baseline, which may explain the difference in the dynamic changes in lower extremity oedema during the hospitalization. Other congestive markers examined in this study showed similar changes from baseline to discharge in women and men.

Prior studies report differing congestive patterns in women and men with AHF with regard to physical examination findings and symptoms at baseline.^2^, ^3^, ^9^, ^10^, ^12^, ^14^

In ADHERE and PROTECT, women with AHF demonstrated more pronounced pulmonary congestion at baseline with a higher prevalence of dyspnoea,^2^ orthopnoea,^3^ and rales,^2^, ^3^ although these differences were subtle in the ADHERE registry. By contrast, in the ALARM‐HF registry and the RELAX‐AHF trial, there were no significant sex differences in measures of pulmonary congestion based on dyspnoea, orthopnoea, or rales at baseline between women and men.^10^, ^12^ In our cohort, LUS findings were similar in women and men, and likewise, there were no significant differences in auscultation findings despite the fact that women were more dyspnoeic.

Regarding the other physical exam parameters, prior AHF trials and data from registries have reported heterogeneous findings with respect to sex differences. While the higher prevalence of peripheral oedema in men in the current study was in line with results from the ADHERE registry,^2^ the RELAX‐AHF and PROTECT trials detected no significant sex differences in the presence of peripheral oedema.^3^, ^12^ However, in the ALARM‐HF registry, more women than men had oedema at baseline,^10^ underscoring the heterogeneous findings of congestion across the different AHF cohorts. The prevalence of jugular venous distension in women and men did not differ significantly in the current study, matching the results of ALARM‐HF, RELAX‐AHF, and PROTECT.^3^, ^10^, ^12^

In terms of the echocardiographic findings, women had better left and right systolic function and higher measures of filling pressures (E/e′). The differences in E/e′ despite similar LUS findings may be explained by a slightly older age in women compared with men in this study, as E/e′ increases with age.^15^ Another potential explanation may be that women had more impaired diastolic function with elevated filling pressures and HFpEF, whereas men had more biventricular systolic HF as indicated by a lower ejection fraction and TAPSE compared with women. This is supported by the larger proportion of men with a prior history of HF and lower extremity oedema and a higher maximum IVC diameter at baseline in men. The higher left ventricular filling pressures (E/e′) in women compared with men in this study is in line with the results of prior studies in both patients with HFpEF and HF with reduced ejection fraction.^16^, ^17^, ^18^

Although previous studies have found that natriuretic peptide levels are higher in women than in men in the general population,^19^, ^20^, ^21^ we did not find a significant difference in NT‐proBNP levels between women and men with AHF at baseline. Prior studies in hospitalized patients with AHF have reported heterogeneous findings with respect to sex differences in natriuretic peptides.^9^, ^11^, ^12^, ^14^, ^22^, ^23^, ^24^ This could be explained by differences in the proportion of patients with atrial fibrillation and worsening renal function across these studies among other factors.

### Outcomes

In accordance with prior studies comparing long‐term outcomes of mortality and/or readmission in women and men with AHF,^9^, ^13^, ^14^, ^22^, ^23^, ^24^ we did not find a significant difference in the risk of the 90 day composite outcome in women compared with men in this study. We found that the risk of the composite 90 day outcome increased with higher number of B‐lines on four‐zone LUS2 in both women and men in an unadjusted, exploratory analysis, highlighting the importance of monitoring and treating pulmonary congestion properly prior to discharge in both women and men. There was no significant interaction between sex and B‐lines on four‐zone LUS. These associations suggest that pulmonary congestion based on the number of B‐lines on LUS has a similar prognostic value in women and men. However, these associations should be investigated further in larger, prospective studies allowing for adjustment for important clinical and demographic variables. There was no significant interaction between sex and any of the examined congestive markers at discharge, suggesting that subtle differences between women and men could be due to a limited number of observations and events and that the congestive markers display an overall similar prognostic pattern in women and men.

Interestingly, only B‐lines on LUS2 but not LUS1 remained important predictors of the composite outcome when both were included in the same model adjusting for sex. Similarly, for dyspnoea scores, only the discharge parameters were significant predictors of outcomes. These findings highlight the importance of pre‐discharge assessment of congestion in patients who are hospitalized for AHF both clinically and in research.

### Strengths and limitations

To the best of the authors' knowledge, this is the first study to specifically investigate sex differences in LUS findings in a cohort of AHF patients. This study employed offline analysis of all LUS videos, temporal blinding during image analysis, and standardized endpoint adjudication. However, because of the limited number of patients with pre‐discharge ultrasound examinations, we were unable to conduct multivariable analyses of the predictors of the 90 day outcome in women and men separately. Therefore, differences in the prognostic importance of LUS findings between women and men should be considered hypothesis generating and investigated further in larger studies. Furthermore, patients did not undergo a pre‐discharge echocardiographic examination or NT‐proBNP measurements in the majority of cases, and therefore, we could not investigate the dynamic changes in these parameters from baseline to discharge or their association with 90 day outcomes.

## Conclusions

Among patients hospitalized for AHF, there were statistically significant sex differences in the echocardiographic parameters at baseline, whereas LUS findings, CXR findings, and NT‐proBNP levels did not differ significantly between women and men. Furthermore, there was a similar and significant decrease in the number of B‐lines on LUS from baseline to discharge in women and men. In an exploratory analysis, the risk of the 90 day composite outcome increased significantly with higher number of B‐lines on four‐zone LUS at discharge in both women and men. These associations should be investigated further in larger studies.

## Conflict of interest

J.D.G. has received research support from Amgen Pharmaceuticals. J.J.V.M.'s employer, Glasgow University, has received payments from Alnylam, Amgen, AstraZeneca, Bayer, Boehringer Ingelheim, BMS, Cardurion, Cytokinetics, Dal‐Cor, GSK, KBP Biosciences, Novartis, Pfizer, and Theracos for his work on clinical trials, consulting and other activities and he has received personal payments from Abbott, Hikma, Ionis, Sun Pharmaceuticals and Servier. S.D.S. has received research support from and personal fees from Alnylam, Amgen, AstraZeneca, Bellerophon, Bristol Myers Squibb, Celladon, Gilead, GlaxoSmithKline, Ionis, LoneStar Heart, Mesoblast, MyoKardia, NIH/NHLBI, Novartis, Sanofi Pasteur, and Theracos and has received personal fees from Akros, Bayer, Corvia, Ironwood, Merck, Pfizer, Roche, Takeda, Theracos, Quantum Genetics, AOBiome, Janssen, and Cardiac Dimensions. E.P.'s employer has received support from Novartis for consulting work, and she has consulted for scPharmaceuticals outside of the submitted work. She has received research support from NHLBI and NIDDK outside of the submitted work. All other authors report that they have no relationships relevant to the contents of this paper to disclose.

## Funding

This work was supported by the National Institutes of Health/National Heart, Lung, and Blood Institute (NIH/NHLBI) (K23HL123533 to E.P.) and the British Heart Foundation (PG/13/17/30050 to R.T.C. and J.J.V.M.). C.E. received a scholarship from the Fulbright Association and from Gentofte Hospital in Denmark.

## Supporting information


**Figure S1.** Consort diagram of the inclusion of patients.
**Figure S2.** Correlation between B‐lines on 4‐zone LUS1 and log (NT‐proBNP) levels in women (n = 130) and men (n = 186). Spearman's correlation coefficient for women: 0.36 (*P* < 0.001) and for men: 0.23 (*P* = 0.002).
**Table S1.** Risk of 90‐day composite outcome (n = 132) in women (n = 53) vs. men (n = 79).
**Table S2.** Predictors of 90‐day composite outcome including congestive parameters at baseline and discharge in same model adjusted for sex (n = 132).
