# Supplementary material for: Sex differences in congestive markers in patients hospitalized for acute heart failure
Source: ESC Heart Fail. 2021 Mar 11;8(3):1784–95. doi: 10.1002/ehf2.13300 (PMC8120385; doi:10.1002/ehf2.13300)
Supplement: Supplementary file 1 — Figure S1. Consort diagram of the inclusion of patients. Figure S2. Correlation between B‐lines on 4‐zone LUS1 and log (NT‐proBNP) levels in women (n = 130) and men (n = 186). Spearman's correlation coefficient for women: 0.36 (P < 0.001) and for men: 0.23 (P = 0.002). Table S1. Risk of 90‐day composite outcome (n = 132) in women (n = 53) vs. men (n = 79). Table S2. Predictors of 90‐day composite outcome including congestive parameters at baseline and discharge in same model adjusted for sex (n = 132). [file EHF2-8-1784-s001.docx]

**Sex differences in congestive markers in patients hospitalized for acute heart failure**

**SUPPLEMENTAL MATERIAL**

**Table of contents**

[**Figure 1.** Consort diagram of the inclusion of patients. 2](#_Toc44169273)

[**Figure 2.** Correlation between B-lines on 4-zone LUS1 and log(NT-proBNP) levels 4](#_Toc44169274)

[**Table 1.** Risk of 90-day composite outcome in women vs. men 5](#_Toc44169275)

[**Table 2.** Predictors of 90-day composite outcome including congestive parameters at baseline and discharge 6](#_Toc44169276)

# **Figure 1.** Consort diagram of the inclusion of patients.


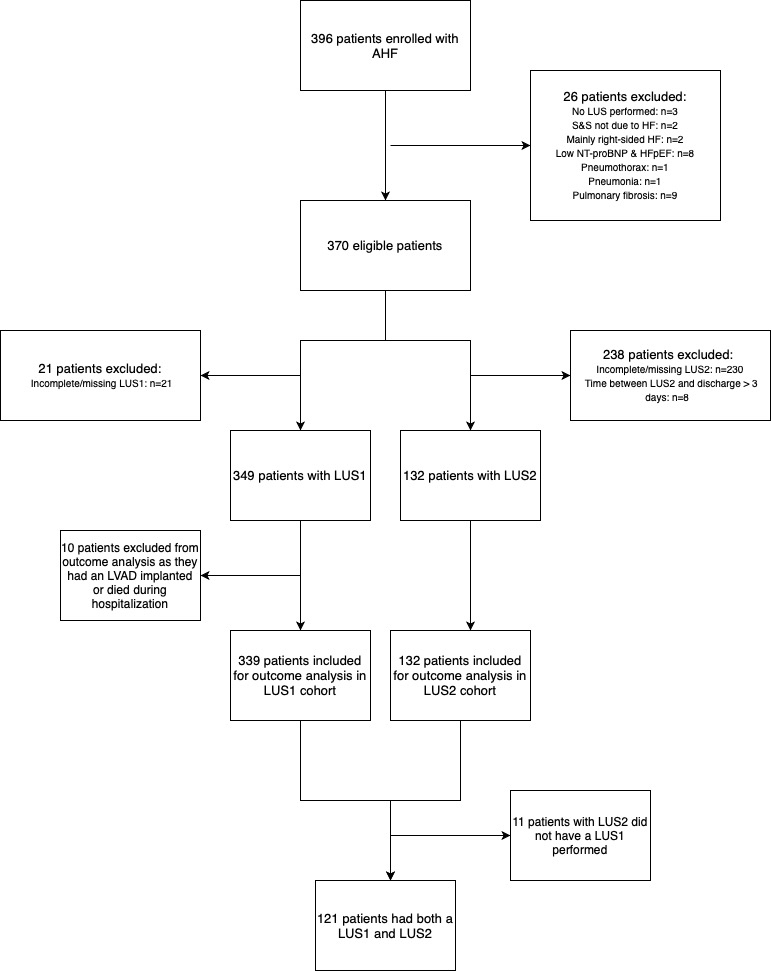


*AHF, acute heart failure; LUS, lung ultrasound; S&S, signs and symptoms; HF, heart failure, HFpEF, heart failure with preserved ejection fraction; LUS1, lung ultrasound at baseline; LUS2, lung ultrasound at discharge; LVAD, left ventricular assist device.*

**Figure 2.** Correlation between B-lines on 4-zone LUS1 and log(NT-proBNP) levels in women (n=130) and men (n=186). Spearman’s correlation coefficient for women: 0.36 (p<0.001) and for men: 0.23 (p=0.002).

**
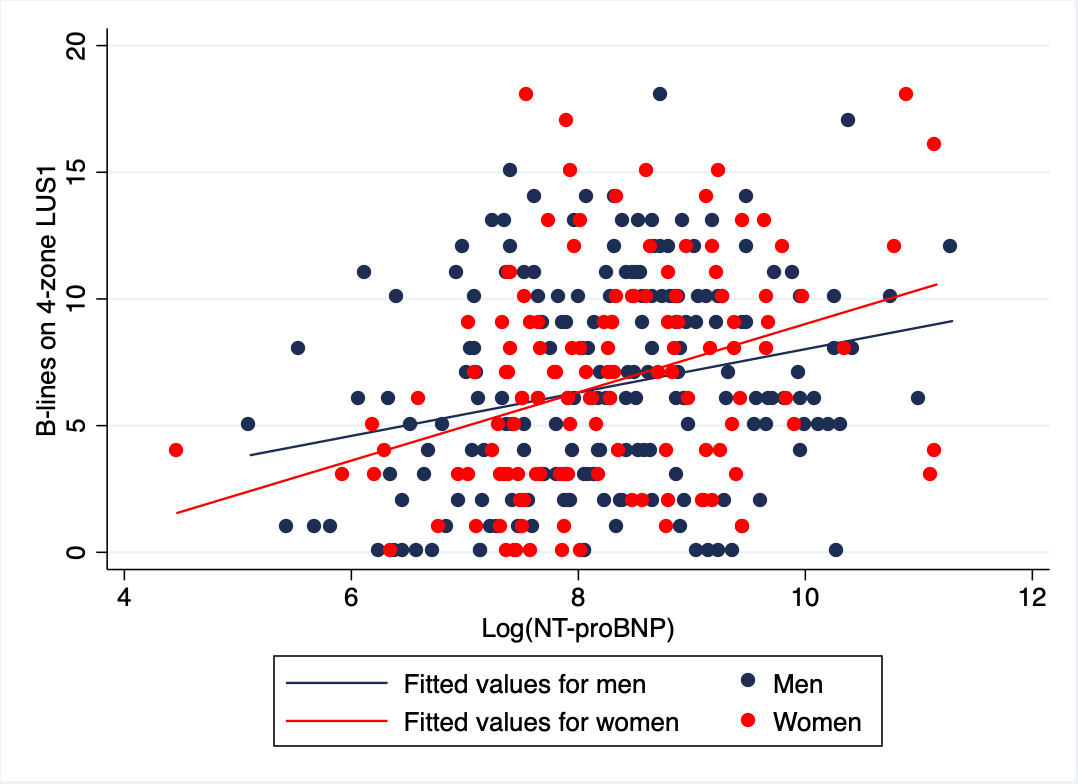
**

**Table 1.** Risk of 90-day composite outcome (n=132) in women (n=53) vs. men (n=79).

|  | **90-day outcome** | |
| --- | --- | --- |
|  | **HR (95% CI) (women compared to men)** | **P** |
| **Unadjusted** | 1.07 (0.58; 1.98) | 0.84 |
| **Model 1*** | 1.87 (0.95; 3.69) | 0.07 |
| **Model 2** † | 1.79 (0.86; 3.74) | 0.12 |

**Model 1: Adjusted for age, left ventricular ejection fraction, baseline log creatinine, and baseline systolic blood pressure, stratified by study site.*

†*Model 2: Adjusted for age, baseline log creatinine, baseline systolic blood pressure, and baseline log NT-proBNP, stratified by study site.*

**Table 2.** Predictors of 90-day composite outcome including congestive parameters at baseline and discharge in same model adjusted for sex (n=132).

| **Congestive parameter** | **Baseline parameter (HR, 95% CI)** | **P** | **Discharge parameter (HR, 95% CI)** | **P** |
| --- | --- | --- | --- | --- |
| ***Dyspnea score (Range 0-10; 10 worst)*** | | | | |
| Dyspnea at rest (per 1 score increase) | 0.90 (0.79; 1.02) | 0.10 | 1.21 (1.07; 1.37) | 0.003 |
| **Physical exam**† | | | | |
| Weight (per 1 kg increase) | 0.92 (0.85; 1.00) | 0.05 | 1.07 (0.99; 1.17) | 0.09 |
| SpO_2_ (per 1 % increase) | 0.96 (0.83; 1.10) | 0.53 | 1.04 (0.89; 1.22) | 0.62 |
| Supplemental O_2_ | 0.83 (0.41; 1.67) | 0.60 | 1.19 (0.42; 3.41) | 0.74 |
| JVD >10cm | 1.27 (0.54; 3.00) | 0.58 | 1.71 (0.69; 4.21) | 0.25 |
| Crackles (any) | 0.80 (0.42; 1.53) | 0.50 | 1.33 (0.69; 2.58) | 0.39 |
| Leg edema (any) | 0.51 (0.25; 1.04) | 0.07 | 1.41 (0.71; 2.82) | 0.33 |
| **Laboratory** | | | | |
| Sodium (per 1 mmol/l increase) | 0.99 (0.92; 1.06) | 0.71 | 0.97 (0.88; 1.07) | 0.53 |
| BUN (per 1 mg/dl increase) | 1.00 (0.98; 1.02) | 0.94 | 1.01 (0.99; 1.03) | 0.18 |
| Creatinine (per 1 mg/dl increase | 1.94 (0.88; 4.28) | 0.10 | 0.71 (0.34; 1.45) | 0.34 |
| **Lung ultrasound** | | | | |
| B-line count (4-zones) (per tertile increase, trend) ‡ | 0.99 (0.92; 1.08) | 0.85 | 1.18 (1.08; 1.29) | <0.001 |
| B-line count (8-zones) (per tertile increase, trend) § | 1.00 (0.95; 1.04) | 0.87 | 1.10 (1.04; 1.15) | 0.001 |
| Pleural effusion score (per tertile increase, trend) \|\| | 0.97 (0.80; 1.18) | 0.75 | 1.08 (0.86; 1.34) | 0.51 |

**SpO_2_, blood oxygen level; Supplemental O_2_, supplemental oxygen, JVD, jugular venous distension; BUN, blood urea nitrogen.*

† *S3 was not included in the outcome analysis, as there were too few measurements for women.*

‡ *B-line tertiles for 4 zone LUS: 0-3, 4-6, ≥7 B-lines.*

§ *B-line tertiles for 8 zone LUS: 0-5, 6-11, ≥12 B-lines.*

|| *Pleural effusion score tertiles; 0, 1-4, 5-8.*
